# The shared-family environmental hypothesis for sex differences in cognitive decline: support from an Italian general population twin study

**DOI:** 10.1007/s10072-026-09075-4

**Published:** 2026-05-20

**Authors:** Corrado Fagnani, Nicola Vanacore, Francesco Sciancalepore, Elisa Fabrizi, Emanuela Medda

**Affiliations:** 1 Italian Twin Registry, Centre for Behavioural Sciences and Mental Health, Italian National Institute of Health (ISS), Rome, 00161 Italy; 2 National Centre for Disease Prevention and Health Promotion, Italian National Institute of Health (ISS), Rome, 00161 Italy

Dear Editor-in-Chief,

We read with extreme interest the review by Cicero et al. [1] on sex and gender-related differences in neurological diseases. It was a pleasure for us to learn that some Italian colleagues in the research community are concerned about a theme that we are also currently tackling, particularly with respect to dementia and cognitive decline. If the review by Cicero and colleagues focuses, among other disorders, on diagnosed Alzheimer’s Disease (AD) in clinical settings, we are more involved in the study of preclinical signs of cognitive deterioration in seemingly unaffected groups, aiming to generate useful evidence for early interventions in asymptomatic or pauci-symptomatic subjects.

Within the ongoing project titled “*From prevention to the etiopathogenetic and pathophysiological mechanisms of dementia: a paradigm shift in the biological continuum of cognitive decline. The PREV-ITA-DEM study*”, funded by the European Union - Next Generation EU, we have the mandate to conduct the first general population twin study in Italy aimed, among other objectives, to estimate the role of genetic and environmental factors in cognitive decline.

The population-based Italian Twin Registry (ITR) [2], in conjunction with the Twin Study Design (TSD) [3], is well suited to accomplish this and the other objectives of the project [4].

As for the estimation of genetic and environmental components of cognitive decline, 520 twins (aged 60–85 years, 63% females) were recruited for the purposes of the above-mentioned project. This sample includes 239 intact pairs [122 monozygotic (MZ), 112 dizygotic (DZ), 5 of unknown zygosity], plus 42 unmatched twins. Cognitive status was assessed by the *Self-Administered Gerocognitive Examination* (SAGE) questionnaire, a first-line tool aimed at flagging potential cognitive concerns; it provides a total score ranging from 0 to 22, with values lower than 17 calling for further clinical evaluation. The tool was previously validated in community-based cohorts and in memory clinics, with more than acceptable performance in terms of sensitivity and specificity for the detection of Mild Cognitive Impairment (MCI) and early dementia [5]; furthermore, it was effectively used in large-scale mail surveys, thus proving to be suitable also in epidemiological contexts where neuropsychological assessment is not feasible.

As a unique natural experiment for the sharing of innate risks and acquired familial tendencies, twins have long been providing significant evidence on the genetic and environmental components of human complex phenotypes, and cognition has been no exception. Based on the assumptions of the TSD, primarily the *Equal Environments Assumption* on the non-differential sharing of trait-relevant exposures for MZ and DZ twins, a higher within-pair correlation – for a given trait – in MZ compared to DZ twins speaks in favour of genetic effects on the trait, while a similar correlation pattern in both zygosity groups, or even a stronger correlation in DZ twins, suggests a key role of family-based influences. This preliminary information can be used to derive genetic and environmental components’ estimates – and related inferential statistics – for the studied phenotype by means of quantitative genetic modelling techniques. More precisely, the phenotype’s variance can be decomposed into additive genetic (“A”), either shared (familial) environmental (“C”) or non-additive genetic (“D”), and unshared (individual-specific) environmental (“E”) components, and their proportions relative to total variance can be calculated [3]. Only three of these four variance components can be simultaneously estimated because C and D components are confounded when analysing data from MZ and DZ twins reared together. We applied ACE rather than ADE modelling as we were mainly interested in comparing the genetic and shared environmental contributions in males versus females. The additive genetic proportion represents the so-called “(narrow) heritability” of the phenotype, while the shared environmental proportion can be regarded as the amount of individual phenotypic differences originating from between-family differences. This latter component of variance is rarely found significant for traits assessed in adulthood, as the effects of family exposures generally vanish over the life course. When this component turns out to be significant in adult samples, its interpretation deserves particular attention, as it could contain the secret of gender differences typically observed for some health outcomes.

Coming to our twin study on cognitive functioning as assessed by the continuous SAGE score, for males we estimated age- and education-adjusted within-pair score correlations of 0.44 in MZ and 0.10 in DZ twins, while for females an opposite pattern emerged, with the corresponding estimates being 0.14 (MZ) and 0.34 (DZ). These correlations were consistent with an “AE” model (i.e., heritability plus individual-specific environmental effects, with no shared environmental influences) for males, and a “CE” model (i.e., familial and individual-specific environmental effects, with no heritable component) for females. In males, “A” and “E” estimates were 0.42 (95%CI 0.11–0.64) and 0.58 (0.36–0.89) respectively, while in females, “C” and “E” estimates were 0.25 (0.08–0.40) and 0.75 (0.60–0.92) respectively.

Such results brought us directly to the issue of sex and gender-related differences in the risk of cognitive decline and dementia, recently tackled by Cicero and colleagues [1] in their *Neurological Sciences* review. As outlined by these authors, one of the most documented aetiological mechanisms for diagnosed AD involves cognitive reserve and resilience. This paradigm refers to individual’s ability to maintain or regain cognitive function despite brain aging, damage, or disease. Among the determinants of this ability, a special mention has to be made to factors like education, occupation complexity, and social engagement. It is well known that in Italy and in many other contexts, the female gender has long been penalised with respect to these factors, due to cultural-based family roles, which have typically provided young women with less extra-family opportunities for medium-to-high education levels, challenging work experiences, or stimulating social interactions, as compared to their male peers. The consequences of these female-specific familial disadvantages may, to date, be detected in women of late-adult and elderly ages, also as a result of cohort effects. Ultimately, this may cause between-family differences to affect individual differences observed in female risk of cognitive decline and dementia, and may also partially mask the genetic origins of this risk.

In our twin study, we had the unique opportunity to verify the above aetiological mechanism of decaying cognition, for the first time in the Italian general (i.e., non-clinical) population. If the mechanism is true, then in a genetically-informed study of MZ and DZ twins, one should be able to detect a significant estimate of the shared environmental component for the targeted cognitive measure; furthermore, a certain degree of underestimation of genetic effects should be expected. This is exactly what we found for the total SAGE score in females: no evidence of heritability emerged, and one-quarter of variance was suggested to originate from the family background, with the lower bound of the 95% CI for the estimate being well above 0, even with a relatively low number of female pairs (82 MZ, 61 DZ).

We are aware that the evidence we provide should be interpreted with caution, due to the limitations of our twin study, mainly including: (i) the self-report nature of data, which raises concerns about completion accuracy; (ii) the tool used for cognitive assessment, which, though previously validated for community screening, is not specifically designed to capture subtle or preclinical cognitive changes; (iii) the modest sample size, which generated wide confidence intervals of variance components estimates in the sex-specific analysis. Furthermore, we should recognise that the interpretation of the shared environmental effects found in females as reflecting sex-related sociocultural disadvantages, though plausible, remains somewhat speculative. Future studies in the Italian elderly - not necessarily twin - population are warranted to better assess the role of retrospectively-measured, family-centred sociocultural factors in sex-specific risk of cognitive decline. On the other hand, the use of a nationally representative, population-based registry for subjects’ recruitment [2], together with the adoption of a powerful and unusual study design [3] should be acknowledged as strengths.

Despite some limitations, our twin study is totally new in the Italian context and further supports a hypothesis on a relevant public health issue, namely sex and gender-related differences in cognitive decline and dementia, whose potential causes are summarised by Cicero et al. in their review [1].
